# Our New York Letter

**Published:** 1890-06

**Authors:** Wm. L. Russell

**Affiliations:** New York


### Correspondence.

### OUR NEW YORK LETTER.

NEW YORK, May 12, 1890.

Expression of the placenta by the Crédé method, or some modification of it, is, it may be safely said, the almost universal custom in the management of the third stage of labor. It can hardly be claimed that Credé was the originator of the idea of expelling the placenta by pressure, for the custom has been found to exist among some uncivilized nations, and in one instance, at least, the lower animals have shown some knowledge of its value. The instance referred to is that of the elephant, "Queen," which was delivered at the winter quarters of Barnum's menagerie a few years ago. After the calf was born, the mother, seeing that the membranes were intact, immediately ruptured them with her paws. Then, as the placenta did not come away spontaneously she placed herself over the short post to which she was chained, and by bringing her weight to bear, actually expressed the after-birth from the vagina. To Cérdé, however, belongs the credit of having introduced the method to the scientific world, and of having stated precise rules for its employment. It has not been allowed to go unchallenged, however, and some very able observers have gone so far as almost to prohibit its use, declaring it to be unphysiological and dangerous. Indeed, it was at one time reported that Crédé himself had abandoned it, but this is now known to have been untrue.

Prof. Wm. T. Lusk, the eminent obstetrician, discussed the subject in a paper read by him at the last meeting of the county Medical Association. He stated that previous to the introduction of the Crédé method, there was a division in the profession in regard to the best plan of procedure, one body advocating that the expulsion of the placenta be left to nature, the other recommending traction on the cord or introduction of the hand. Between 1846 and 1848, Crédé had gradually developed his method, having begun by merely keeping his hand on the abdomen for the purpose of observing the conduct of the uterus after the birth of the child. The custom now was to wait fifteen or twenty minutes, when the ptacenta had in most cases descended into the lower segment of the uterus, and, then, during a contraction, to make firm pressure in the direction of the axis of the superior pelvic strait. The placenta could, in the large majority of cases, be in this way expressed from the vagina. Roughness should be carefully avoided. The opponents of this method claimed that it caused the loss of a larger amount of blood than would otherwise occur, and that there was more risk of septicæmia from retention of the membranes. There was no ground for these objections if the procedure was properly conducted. When the placenta had reached the vulva, no traction should be made on the membranes while the uterus remained hard. The placenta should under these circumstances be supported by the hand until the uterus relaxed, when the membranes could be safely withdrawn. The only questions to be decided were whether the method was physiological, and the time after the child's birth at which it should be adopted. In the discussion which followed the reading of the paper, a former pupil of Crédé remarked that Crédé's direction was to press toward the hollow of the sacrum so as to flatten the uterus rather than press it downwards.

Dr. George T. Harrison, the President of the Association, said that, in regard to the question of the time to make pressure, it was his custom to note the rise of the uterus from the pelvis. This was coincident with the descent of the placenta below the ring of contraction, and it was then that pressure should be made, the contracting portion of the uterus being pressed downward in the flabby segment below. In closing, Prof. Lusk added that there was nothing that he loved so much to teach the young obstetrician as this method of delivering the placenta. He himself was able to effect delivery in nearly every case after at most three or four attempts during as many pains. If he did not then succeed he would introduce his hand.

In my first letter I referred to a paper read before the Academy by Dr. H. C. Coe, in which he claimed that the operation of laparotomy for disease of the uterine appendages was in many cases not only unsuccessful in relieving the symptoms which led the patient to consent to it, but was productive of positive harm from adhesions forming about the stump, and from the mental disorders which were of by no means infrequent occurrence. The subject was still further discussed at the last meeting of the Section on Obstetrics and Gynecology. Dr. H. J. Bolat came to the defence of the operation with a record of a hundred and twelve cases, of whom fifty-eight were found after two years to have completely recovered from all symptoms. He was warmly supported by Dr. Joseph Price, of Philadelphia, who stated that he had seen no cases of insanity caused by the operation, while he had known several instances in which mental troubles had been cured. He regarded hospitalism which so frequently resulted from prolonged treatment as equally bad. He believed that inflammatory sequelæ, such as had been described in Dr. Coe's papers, were the result of poor execution. On the other hand he had seen cases in which inflammatory trouble existed in the pelvis, where phthisis was threatened, in fact where cough and hemorrhage had already appeared, in which complete cure resulted after operation. In eight cases of puerperal fever, where death seemed inevitable, he had seen the operation followed by relief.

On the other side of the question, Dr. Lusk stated that he had seen several instances of mental depression following the operation in young women. Dr. Wm. M. Polk supported this statement, and added that he had lately been trying a waiting plan of treatment in such cases as he had formerly operated on. His experiments had not yet been sufficiently prolonged to enable him to come to a decision. He had also opened tubes and ovaries, washed them out and returned them, with no bad results thus far.

At the same meeting Dr. C. D. Scudder reported a case of labor occurring in a woman with a kyphotic pelvis, where preparations had been made for Cæsarean section, but in which, much to the surprise of all, delivery was effected by version. Several other gentlemen then related histories of similar cases in which natural delivery had occurred under very difficult conditions. In one case, the vagina was so small as to admit with difficulty one finger. The girl was only thirteen years old, with an extremely small pelvis, and almost doubled from distortion of the vertebral column. It was thought that operative interference was inevitable. Labor began, however, before the arrangements had been completed, and a living child of good size was born with comparative ease, and without causing any laceration of the soft parts. Another striking case was referred to in which, in a woman with a small pelvis, it had been thought necessary to interfere in various ways in her first four pregnancies, so that none of the children were born alive. In the fifth, however, the induction of premature labor having been decided upon, the woman failed to appear for operation, and at full term succeeded in delivering herself of her first living child. A number of gentlemen participated in the discussion, and the general opinion seemed to be that although the mortality from Cæsarean section had been reduced as low as eight per cent., yet the operation should not be performed till after labor had begun, for it was impossible to predict the amount of compression which the child's head would undergo.

At the last meeting of the Society of Medical Jurisprudence and State Medicine, the subject under discussion was, "The Legal and Medical Aspect of Drunkenness." This society is composed of a number of the ablest lawyers and physicians in the city, and the discussions are usually on live topics and full of interest. The paper of the evening was read by Judge McAdam. He set forth the necessity ofa State institution where indigent inebriates could be confined, humanely cared for, and, if possible, cured and restored to usefulness. The questions offered for discussion were : (1) Is a person who gets drunk habitually afflicted with disease ? (2) Does the public weal demand that such individuals be sent to jail as criminals, or to sanitariums as patients? The opinion of the physicians who spoke seemed to be that in a certain proportion of drunkards the habit could be traced to disease. Such cases were those classed as dipsomaniacs, who had inherited a tendency to intemperance, and were subject to a periodical, irresistible craving, which had to be satisfied ; also those in whom the habit had been engrafted on an organization already weak from congenital feebleness of will power, or deficiency in brain development. The latter as a rule desired to reform, were certainly susceptible of cure if placed under favorable conditions, and ought not be classed as criminals.

During a recent clinic at the Post-Graduate school, Dr. Daniel Lewis, of the Skin and Cancer Hospital, gave the following formula of what he regarded as the most satisfactory dressing for foul cancerous ulcers:

℞ Cretæ Preparatæ.... ..................℥ss.

Ol. Amygdal. Dulcis........................ʒi.

Lanolini..................................℥i.

Ol. Bergamot..........................gtt. vii.

*M* WM. L. RUSSELL.

